# Prediction of the adherence to sports practice of young Ecuadorians based on the perception of the coach’s interpersonal style

**DOI:** 10.3389/fpsyg.2023.1133583

**Published:** 2023-04-26

**Authors:** Diego Andrés Heredia-León, David Manzano-Sánchez, Alberto Gómez-Mármol, Alfonso Valero-Valenzuela

**Affiliations:** ^1^Department of Physical Activity and Sports, Faculty of Sport Sciences, SAFE (Salud, Actividad Física y Educación) Research Group, University of Murcia, Murcia, Spain; ^2^Academic Unit of Education, Catholic University of Cuenca, Cuenca, Ecuador; ^3^Faculty of Education and Psychology, University of Extremadura, Badajoz, Spain; ^4^Faculty of Education, SAFE (Salud, Actividad Física y Educación) Research Group, University of Murcia, Murcia, Spain

**Keywords:** autonomy support, basic psychological needs, motivation, intention to be physically active, sport

## Abstract

Based on the framework of the self-determination theory, the present study aimed to test a predictive model in the Ecuadorian sports context by using autonomy support as a trigger, which was mediated by basic psychological needs and later by autonomous motivation. This procedure was used to predict the intention to be physically active and was carried out on 280 athletes from the province of Azuay (Ecuador) aged between 12 and 20 years (*M* = 15.28; SD = 1.71). Different scales were used to measure the perception of the interpersonal style of autonomy supported by the coach. The scales employed included the degree of satisfaction of basic psychological needs, motivation to practice sports, and intention to be physically active. A structural equation analysis revealed that perceived autonomy support positively predicted basic psychological needs, which in turn positively predicted autonomous motivation and, as a result, the athletes’ intentions to be physically active. It was concluded that coaches’ support for the interpersonal style of autonomy can benefit the development of basic psychological needs as well as autonomous motivation, which in turn can increase young athletes’ intentions to be physically active. Future research is also recommended to verify this predictive model and to encourage further experimental studies in which coaches promote autonomy support for athletes intending to increase their adherence to practising sports.

## 1. Introduction

Regular physical activity and sports are beneficial for physical health and for the mental health of children and adolescents. However, a large part of the population is becoming less and less active due to changes in lifestyle patterns, the use of transportation, the increasing use of technology, changing cultural values, and urbanization (World Health Organization, 2018). Despite the possible advantages that can be obtained by practising sports, dropout rates indicate that half of the children and adolescents who start an activity end up quitting during the first 6 months (Molinero et al., 2011); hence the interest in studying variables that help improve the population’s intention to practice a physical activity. One of these variables is motivation, which denotes someone’s energy and direction as well as the persistence of and purpose behind their behaviors, including their intentions and resulting actions, thus placing itself at the center of the biological, cognitive, and social regulation of the individual (Deci and Ryan, 1985).

In order to understand motivation, Self-determination theory (SDT) (Deci and Ryan, 1985, 2000) is one of the most popular contemporary frameworks that describes the process through which it develops and how it influences human behavior and wellbeing. Deci and Ryan (1991) postulated that the regulation of human behavior could be framed in terms of autonomous, controlled, or lack of motivation. In SDT, autonomous motivation is defined as the participation in activities for the interest and satisfaction derived from the activities themselves (Hagger et al., 2014). This motivation consists of intrinsic (the individual performs an activity because it is fun and attractive), identified (the individual identifies with the value of the activity and has a predisposition to act), and integrated motivation (the individual finds the activity congruent with his/her values and interests). Embedded within SDT, the basic psychological needs are fundamental to increase a more autonomous motivation and for that, positive consequences (Vallerand, 1997). These basic psychological needs are autonomy, competence and relatedness. Autonomy can be defined as one’s need to experience a sense of willingness in one’s actions. Competence refers to one’s need to experience effectiveness in one’s interactions with the world. Finally, relatedness refers to a need for connectedness with significant others, satisfaction with the social world, and a feeling of being accepted (Ryan and Deci, 2017).

From this perspective, coaches should consider the importance of supporting the autonomy of their athletes since it can create a context in which athletes feel their decisions are voluntary (autonomy perception). It follows that their behaviors would take on a more intrinsic character at the motivational level (Ryan and Deci, 2017) and according to studies like Merino-Barrero et al. (2019) or Rodrigues et al. (2019) it could predict higher levels of the intention to continue exercising in the future.

This theory has been investigated through different studies in recent decades, including those related to sports, exercise, and physical education. For instance, some studies have attempted to predict the intention to be physically active through different variables such as the interpersonal style of the coach/teacher (Almagro and Conde, 2012; Expósito et al., 2012; Baena-Extremera and Granero-Gallegos, 2015; Fernández-Ozcorta et al., 2015; Moreno-Murcia et al., 2018; Cid et al., 2019; Trigueros-Ramos et al., 2019; Huéscar et al., 2020), the satisfaction of basic psychological needs of autonomy, competence and relatedness (Almagro and Conde, 2012; Fernández-Ozcorta et al., 2015; Franco et al., 2017; Rodrigues et al., 2020) and autonomous motivation (Lim and Wang, 2009; Méndez-Giménez et al., 2012; Cuevas et al., 2014; Franco et al., 2016; Rodrigues et al., 2019; Teixeira et al., 2020). Focusing particularly on the sports field and based on the importance of autonomy support teaching style from coach to athlete (Deci and Ryan, 1987), numerous studies have focused on this variable since it demonstrates high predictive power in relation to the satisfaction of the basic psychological needs (Isoard-Gautheur et al., 2012; Morillo et al., 2018; Pineda-Espejel et al., 2020).

Various studies have analyzed these variables separately since no studies in the bibliography have tested the SDT’s complete sequence—support for autonomy, basic psychological needs, and autonomous motivation—using intention to be physically active as the final variable.

For this reason, based on the SDT, this study aimed to test a model in relation to Ecuadorian athletes using structural equations where the predictive power of the perception of the coach’s interpersonal style was seen as supporting autonomy in terms of basic personal needs along with autonomous motivation and, finally, autonomous motivation to continue to be physically active in the future. It was deemed very important to assess the intention to be physically active in this age range because practising sports is usually interrupted by the start of higher education since in Ecuador many students do not have access to a public university and have to work to pay for their studies (Almagro et al., 2011). Following this, it was hypothesized that the interpersonal style of a coach that supports autonomy would predict the satisfaction of basic psychological needs, which in turn would predict autonomous motivation and, finally, the intention to be physically active.

## 2. Materials and methods

### 2.1. Design

This research corresponds to a cross-sectional explanatory design, meaning that an empirical study is considered alongside an associative strategy, where theoretical models are put together to test their integration into an underlying theory (Ato et al., 2013).

### 2.2. Participants

In the present study, the sample comprised 301 federated Ecuadorian athletes in the province of Azuay. The sample selection was a non-probabilistic type for the sake of convenience according to the subjects that could be accessed. After discarding the questionnaires that were not fully completed and applying statistical procedures to detect atypical cases and missing values, the final sample comprised 280 male (*n* = 153) and female (*n* = 127) athletes aged between 12 and 20 years, with the mean age being 15.28 (SD = 1.71).

### 2.3. Measures

#### 2.3.1. Support autonomy

Support for autonomy was assessed using seven items belonging to the “Support for Autonomy Scale” developed by Moreno-Murcia et al. (2020). Participants had to answer questions about the interpersonal style of the coach in practices aimed at supporting autonomy (e.g., “offers different ways to perform a certain task”). The previously used statement was the following: “In my training sessions, my coach …” The scale corresponds to a Likert-type scale, with five response options from one (surely not) to five (surely). The internal consistency coefficients showed a value α = 0.75 and Ω = 0.76.

#### 2.3.2. Basic psychological needs

The “Psychological Need Satisfaction in Exercise Scale” (PNSE) developed by Wilson et al. (2006) was validated for the Spanish context by Moreno-Murcia et al. (2011). The PNSE uses 18 items, six of which assess each of the following needs: competence (e.g., “I have the confidence to do the most challenging exercises”), autonomy (e.g., “I believe that I can make decisions regarding my exercise program”), and relatedness (e.g., “I enjoy camaraderie with my classmates because we exercise for the same reason”). The previous sentence started with “In my training …,” and the responses were collected on a Likert-type scale, whose score ranges from one (false) to six (true). Internal consistency demonstrated a value of α = 0.84, competence Ω = 0.84, autonomy α = 0.76 and Ω = 0.76, and relatedness α = 0.66, and Ω = 0.70. In addition, the Psychological Mediators Index (IMP) was developed by calculating the average of the three factors in a single dimension; the internal consistency was α = 0.79 and Ω = 0.81.

#### 2.3.3. Autonomous motivation

The factors that make up autonomous motivation in the “Behavior Regulation Questionnaire in Sport” (BRQS) developed by Lonsdale et al. (2008) were validated for the Spanish context by Moreno-Murcia et al. (2011). Six factors from four items were used to measure general intrinsic motivation (e.g., “because I find it pleasant”), intrinsic motivation for knowledge (e.g., “because I enjoy learning new techniques”), intrinsic motivation for stimulation (e.g., “because of the pleasure it gives me when I am fully dedicated to this sport”), intrinsic motivation for achievement (e.g., “because it gives me satisfaction when I strive to achieve my goals”), integrated regulation (e.g., “because practising this sport is part of who I am”), and identified regulation (e.g., “because it is a good way to learn things that can be useful in my daily life”). The introductory phrase used was the following: “I participate in this sport …” A seven-point Likert scale was used, ranging from one (totally false) to seven (totally true). Following Williams et al. (1996), to analyze the data in the present work we unified the four dimensions of intrinsic motivation along with integrated regulation and identified regulation into a single dimension called autonomous motivation. The reliability of the variables for the athletes was α = 0.61 and Ω = 0.67 for general motivation; α = 0.78 and Ω = 0.77 for intrinsic motivation for knowledge; α = 0.71 and Ω = 0.72 for intrinsic motivation for stimulation; α = 0.75 and Ω = 0.76 for intrinsic motivation for achievement; α = 0.77 and Ω = 0.78 for integrated regulation; α = 0.67 and Ω = 0.69 for identified regulation; and α = 0.84 and Ω = 0.84 for the unified factor of autonomous motivation.

#### 2.3.4. Future intention to be physically active

The questionnaire Intention to be physically active (IPA) was used by Hein et al. (2004) and validated for the Spanish context by Moreno et al. (2007). This tool comprises five items, e.g., “after graduation, I would like to be physically active,” that are preceded by a statement, in this case, “Regarding my intention to practice some physical sporting activity …” The answers are closed with a Likert-type scale whose score ranges from totally disagree (1) to totally agree (5). The reliability value was α = 0.75 and Ω = 0.77.

### 2.4. Procedure

This project was presented to the Azuay Sports Federation in Cuenca–Ecuador, from which permission was also requested. Contact was established with the managers, coaches, and monitors responsible for the participating federation through an informative meeting to inform them of the objectives of the project and request their collaboration and the approval of the representatives of the sports clubs. Underage athletes were asked for written authorization from their parents, guardians, or legal representatives, and once the relevant informed consent was obtained, information was given the athletes on how to fill out the questionnaires and resolve all doubts that may arise during the process. The instruments were administered with the researcher present to provide a brief explanation of the objective of the study, and the questionnaires were administered at the beginning of the training sessions in the different sports venues following a prior agreement with the relevant coach and without his/her presence. The anonymity of the answers was asserted. The time required to complete the questionnaire was approximately 15 min, and it varied slightly according to the age of the athletes. The design was approved by the Research Ethics Commission of the University of Murcia, code 3023/2020.

### 2.5. Data analysis

Following a reliability analysis of all the scales, the Mahalanobis distance was used to detect and eliminate atypical cases or those that did not follow a logical pattern in the set of variables. In addition, the skewness and kurtosis values were analyzed and considered adequate at <2 and <7, respectively (Curran et al., 1996). After eliminating 21 participants who did not meet these requirements, we again proceeded to the reliability analysis of Cronbach’s alpha and McDonald’s omega of the different scales and finally ended up with a total sample of 280 subjects. The McDonald’s omega was calculated following the recommendations of Hayes and Coutts (2020), who consider that this reliability analysis has been more feasible and more widely used in recent years since it works with factor loadings, which makes the calculations more stable and presents a greater level of reliability. The reliability coefficients revealed values for most of the variables above 0.70, a criterion considered acceptable for psychological domain scales (Nunnally, 1978). For the general motivation and regulation identified, the alpha and omega coefficients fell in the range between 0.60 and 0.70, which is also considered acceptable by authors such as Sturmey et al. (2005).

Since this was the first time these instruments were applied to a group of Ecuadorian athletes, a confirmatory factor analysis (CFA) was performed to test the factorial structure of each scale. The findings show that all the rankings exhibited were acceptable with significant factor loadings. Subsequently, the structural equation model was used while considering the same dependent and independent variables (Kline, 2011).

As for the analysis, the following absolute and incremental indices were calculated: Comparative Fair Index (CFI), Tucker-Lewis Index (TLI), and the Root Mean Squared Approximation Error (RMSAE), with their respective confidence intervals (90% CI). The following cut-off points were considered acceptable: CFI and TLI ≥0.90, and RMSEA ≤0.80, following various recommendations (Marsh et al., 2004; Hair et al., 2014). To calculate the direct and indirect effects between the constructs, a 95% Confidence Interval (95% CI) was used, with the CI being significant where zero was not included. Subsequently, the composite reliability coefficient (CR) was calculated while considering values greater than 0.70 (Bagozzi and Yi, 1988; Hair et al., 2014) and mean-variance extracted (AVE), which is satisfied if it is >0.50 (Fornell and Larcker, 1981); however, this criterion can be considered very conservative, and slightly lower values can be accepted (Hair et al., 2014). Finally, the measurement invariance between the two samples was reviewed according to gender, meaning that the configure, metric, scale, and strict invariance were evaluated (Byrne, 2016) while the CFI variation was taken as a criterion (ΔCFI < 0.01) (Cheung and Rensvold, 2002). All analyses were carried out using the statistical package SPSS 25.0 and Amos 23 (SPSS Inc., Chicago, IL, USA).

## 3. Results

### 3.1. Preliminary analysis and bivariate correlations

Table 1 shows the bivariate correlations between variables. All the variables were directly and positively correlated with each other (*p* < 0.001) except for the variable “interpersonal style of autonomy support” with the variable “intention of being physically active” (*p* = 0.52).

**TABLE 1:** Mean, standard deviation, reliability, and bivariate correlations of the study variables.

Constructs	M	SD	A	K	α	Ω	1	2	3	4
Autonomy style	4.28	0.56	−0.93	0.85	0.75	0.76	–	–	–	–
BPN	4.74	0.60	−0.49	0.16	0.79	0.81	0.384*	–	–	–
Autonomous motivation	6.39	0.58	−1.32	1.49	0.84	0.84	0.826*	0.361*	–	–
IPA	4.56	0.56	−0.95	0.40	0.75	0.77	0.091	0.175*	0.266*	–

*p < 0.01; M, media; SD, standard deviation; A, asymmetry; K, kurtosis; α, Cronbach’s alpha value; Ω, McDonald’s omega value; BPN, basic psychological needs (composite factor); IPA, physical activity intention.

### 3.2. Measurement model

Testing the measurement model involved addressing the autonomy-supportive interpersonal style, the satisfaction of basic psychological needs, autonomous motivation (intrinsic motivation, integrated regulation, and identified regulation), and intention to be physically active. Due to the low factorial loads of some of the items that made up the autonomy support scale, and with the intention to present a better fit of the model, the items that were included for the autonomy support factor were all included except 4, 9, 10, and 11. The results showed a good fit for the data [*X*^2^ = 194.326 (126); SRMR = 0.061; RMSEA = 0.044 (90% LO = 0.031, HI = 0.056); TLI = 0.932; CFI = 0.944].

### 3.3. Structural model

**FIGURE 1:**
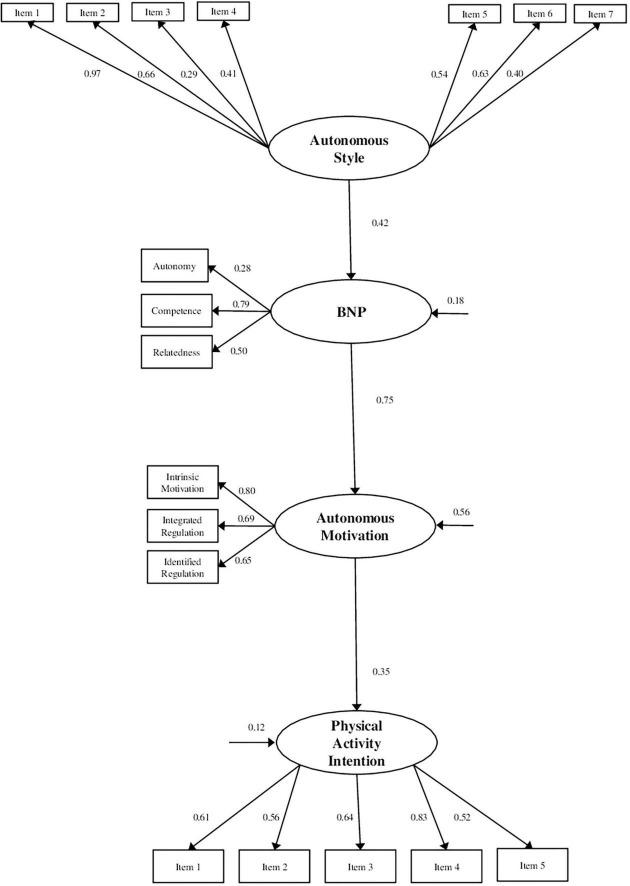
Standardized solution of the structural model of autonomy support, autonomy satisfaction, relationship and competence, autonomous motivation, and the intention to be physically active. All parameters are standardized and significant at *p* < 0.05.

The structural model demonstrated a good fit for the data [*X*^2^ = 189.734 (118); SRMR = 0.058; RMSEA = 0.047 (90% LO = 0.034, HI = 0.059); TLI = 0.911; CFI = 0.932]. For the standardized direct effect (Figure 1), positive and significant associations were observed between all the constructs. Specifically, the association between an interpersonal style of autonomy support and the satisfaction of basic psychological needs (β = 0.42), autonomous motivation (β = 0.75), and autonomous motivation and the intention to be physically active (β = 0.35) were all significant, whereas the indirect effect of autonomy support to intention was β = 0.22 and of basic psychological needs to intention β = 0.06.

### 3.4. Convergent and discriminant validity

Once the confirmatory factorial analysis was carried out, the resulting values of the factors were taken to calculate the convergent validity, thereby fulfilling the latter since all the saturations of the standardized factorial loads yielded values above the 0.50 criterion point. Meanwhile, Table 2 shows the composite reliability (CR) with values greater than 0.70, bar the value of basic psychological needs (0.461). The average variance extracted (AVE) was also evaluated and values above 0.5 were obtained, although the autonomy support style presented a somewhat lower value (0.354).

**TABLE 2:** Convergent and discriminant validity of the model.

Constructs	CR	AVE
Autonomy style	0.788	0.354
BPN	0.461	0.523
Autonomous motivation	0.808	0.641
Physical activity intention	0.744	0.520

BPN, basic psychological needs (composite factor); CR, composite reliability; AVE, average variance extract.

### 3.5. Factorial invariance according to gender

Since the structural model presented a good fit for the data, multigroup confirmatory factor analysis was used to verify its factorial invariance according to gender. First, the configured model (M1) was established and from this basis a series of nested models (M2, M3, and M4) were tested. As can be seen in Table 3, the first model (the M1 base model) did not present restrictions based on gender, thereby showing acceptable fit indices, which allowed for the examination of the configural invariance between groups based on gender. The existence of non-significant differences between the models meant that the constraints could assume invariance across groups. In M2, the factorial weights were restricted to being the same in both groups, and the fit indices were adequate. Therefore, the criterion of metric invariance could be assumed. The findings suggest that the factorial weights were invariant between groups.

**TABLE 3:** Measurement invariance of the model based on gender.

Model	*X* ^2^	gl	Δ *X*^2^	Δ gl	CFI	RMSEA	Δ CFI	Δ RMSEA
**Gender**								
Man	154.912	126	–	–	0.963	0.039	–	–
Woman	189.625	126	–	–	0.864	0.064	–	–
M1	344.587	252	–	–	0.926	0.036	–	–
M2	355.600	266	11.01	14	0.928	0.035	0.002	0.001
M3	382.734	276	27.13	10	0.914	0.037	0.014	0.002
M4	433.603	297	50.87	21	0.890	0.041	0.024	0.004

M1 = Configure, M2 = Metric, M3 = Scale, and M4 = Strict.

In M3, the covariances between the factors were restricted to being the same between the groups, the fit indices of the model were not entirely adequate (ΔCFI > 0.01), differences existed in the covariances between the factors, and the scalar invariance criterion of accuracy could not be assumed. This implies that the strict invariance of the groups, measured through the residual measurement loads (M4), must also be ruled out.

## 4. Discussion

The objective of this study was to explore the predictive power that autonomy support has on basic psychological needs using a sample of Ecuadorian athletes, some of whom have autonomous motivation and/or autonomous motivation with the intention to continue to be physically active in the future. It was hypothesized that the perception of the coach’s autonomy-supportive interpersonal style would act as a positive predictor of the satisfaction of basic psychological needs, which would act as positive predictors of autonomous motivation, while the intention to be physically active would be predicted by the autonomous motivation.

In terms of the first postulate of the model, the results confirm the first part of the hypothesis, thus showing that the autonomy support given by coaches positively predicts the satisfaction of the three basic psychological needs in young Ecuadorian athletes, that is, competence, autonomy, and relatedness. These results have been corroborated in several investigations that have uncovered a positive relationship between autonomy support and the satisfaction of basic psychological needs, such as the case of Adie et al. (2008) in the United Kingdom with 539 participants who practiced different sports and trained an average of 7 h per week; the study by Álvarez et al. (2013) in Spain on 159 Spanish taekwondo athletes; the investigation carried out by González et al. (2015) into 434 Spanish soccer players who belonged to 24 different teams; and the study of López-Walle et al. (2012) in Mexico with 669 athletes from 18 different sports. Therefore, it has been highlighted that the coaches who interacted with their athletes while supporting their autonomy during practice allowed for greater satisfaction of each of the three basic psychological needs, as mentioned by the SDT postulates (Deci and Ryan, 1985, 2000). Huéscar et al. (2020) presented similar findings, indicating the presence of a positive and significant relationship between teacher-induced support for autonomy and the satisfaction of basic psychological needs in sports science students.

On the other hand, and following the sequence of the proposed model, the findings of the present study suggest that basic psychological needs are an essential ingredient for autonomous motivation since athletes who perceive this sequence obtain a greater degree of self-determined motivation. This has been corroborated by different studies from other investigations in the sports and Hispanic-Latino context, where the significant relationship between basic psychological needs and autonomous motivation, self-determined motivation, or intrinsic motivation has been demonstrated. This is the case in the Spain-based studies of Almagro et al. (2011), who analyzed 580 male and female participants who practiced competitive sports, and Balaguer et al. (2008), who studied 301 athletes of different modalities who practiced more than 4 days a week and had been competing for an average of 8 years. Along the same lines, a study was carried out in Mexico by Cantú-Berrueto et al. (2016) with soccer players who had been practicing the discipline for more than two and a half years, while the study by Teixeira et al. (2020) investigated 799 elite swimmers between the ages of 12 and 20 who had an average training experience of 8 years. Finally, in Spain and in the context of physical education, Valero-Valenzuela et al. (2021) carried out a study with a sample of 618 students and obtained results in line with those from the current study. From these results and those of the present study, the contribution of the coach to meeting the basic psychological needs of his/her athletes to achieve optimal autonomous motivation, as mentioned by Deci and Ryan (2000) in their well-known SDT, stands out.

From the third and last postulate of the studied model, it is evident that autonomous motivation is positively related to the intention to be physically active. This relation is of vital importance since in different sports environments motivation may be the most important factor for maintaining fitness in the future (Ryan and Deci, 2007). These statements coincide with the results obtained in various investigations, such as the studies by Almagro et al. (2010) mentioned above; Cid et al. (2019) on Portuguese students who practiced extracurricular sports; Fernández-Ozcorta et al. (2015) on Spanish participants who, as inclusion criteria, had to demonstrate their physical activity; and Lim and Wang (2009) on Singaporean students. Although a great novelty, it is noteworthy that to date no study has predicted the intention of athletes to continue to be physically active, a sequence that has been considered in the present study. If it has been investigated, it is from the opposite point of view, namely, in terms of how the frustration of basic psychological needs predicts the validity of SDT. Indeed, Rodrigues et al. (2019) analyzed athletes who went to gyms and health clubs and found that the frustration of basic psychological needs promoted more controlling motivation and a smaller intention to be physically active. Therefore, it is important to encourage the fulfilment of these three basic psychological needs in order to promote healthy habits and adherence to regular physical activity.

One of the limitations of this research is its cross-correlational nature and the fact that the relationships described do not indicate a causal relationship. Despite this, the study has the following strengths: the consideration of a large sample of federated athletes and the high level of reliability obtained from the instruments together with the adequate values of the predictive model. Consequently, the research provides important information concerning the study variables that stimulate the physically active practice of sport, which reduces problems such as obesity and sedentary lifestyles that are so relevant today. Ideally, longitudinal intervention research into coaches with experimental or quasi-experimental designs could be carried out as already described, such as those that have been carried out in the educational and sports fields in relation to teachers (Serrano et al., 2018; Moreno-Murcia et al., 2019). Similarly, interventions that encourage athletes to intend to continue being physically active have been proposed in other studies with different intervention variables (Martínez et al., 2016; Zamorano et al., 2021). Following the postulates of Vallerand (1997), studies have been proposed to find out if athletes, who are encouraged to support autonomy for a certain time and satisfy their basic psychological needs, develop more autonomous motivation and are physically more active than athletes who are not involved in the intervention group. New studies should also be carried out using different methodological designs, random sampling, and different age groups.

## 5. Conclusion

In conclusion, coaches’ support for autonomy is a fundamental aspect of autonomous motivation due to its relationship with basic psychological needs: these could act as mediators that increase autonomous motivation and thus produce an increase in the intention to be physically active. In short, it is important to focus on the training of coaches to provide them with strategies that favor autonomy support so that athletes can satisfy their different psychological needs, which can in turn motivate young athletes to increase their physical activity and commitment to sports in the future.

## Data availability statement

The raw data supporting the conclusions of this article will be made available by the authors, without undue reservation.

## Ethics statement

The studies involving human participants were reviewed and approved by the Ethics Committee of the University of Murcia (2871/2020). Written informed consent to participate in this study was provided by the participants’ legal guardian/next of kin.

## Author contributions

DH-L, AV-V, DM-S, and AG-M: conceptualization, methodology and investigation, formal analysis, data curation, and writing—review and editing. DH-L: writing—original draft preparation. AV-V: visualization. AV-V, DM-S, and AG-M: supervision. All authors have read and agreed to the published version of the manuscript.
